# The Impact and Burden of Chronic Rhinosinusitis with Nasal Polyps on Patients and Their Family Caregivers: A Nationally Representative Survey

**DOI:** 10.3390/healthcare13040430

**Published:** 2025-02-17

**Authors:** Luca Malvezzi, Veronica Seccia, Antonio Moffa, Frank Rikki Mauritz Canevari, Ilaria Baiardini, Simona Barbaglia, Mattia Battistini, Eleonora Cantoni, Filippo Cipriani, Marta Pirronello, Giovanna Sala, Annalisa Stassaldi, Eugenio De Corso

**Affiliations:** 1 Department of Biological Sciences, Humanitas University, Via Rita Levi Montalcini 4, Pieve Emanuele, 20090 Milan, Italy; luca.malvezzi@humanitas.it; 2 Otorhinolaryngology Unit, IRCCS Humanitas Research Hospital, Via Manzoni 56, Rozzano, 20089 Milan, Italy; 3 Otorhinolaryngology Head & Neck Surgery Unit, Casa di Cura Humanitas San Pio X, Via Francesco Nava 31, 20159 Milan, Italy; 4 Otolaryngology, Audiology and Phoniatric Operative Unit, University Hospital of Pisa, 56126 Pisa, Italy; veronicaseccia@gmail.com; 5 School of Medicine, Università Campus Bio-Medico, 00128 Rome, Italy; 6 Integrated Therapies in Otolaryngology, Fondazione Policlinico Campus Bio-Medico, 00128 Rome, Italy; 7 DISC Department, University of Genoa, 16132 Genoa, Italy; frank.canevari@unige.it; 8 Unit of Otorhinolaryngology-Head and Neck Surgery, IRCCS Ospedale Policlinico San Martino, 16132 Genoa, Italy; 9 Department of Internal Medicine and Medical Specialties, DiMI, University of Genoa, 16126 Genoa, Italy; ilaria.baiardini@libero.it; 10 Associazione Nazionale Pazienti Respiriamo Insieme-APS, 35131 Padua, Italy; presidente@respiriamoinsieme.org; 11 Ethics Committee for Clinical Experimentation LOMBARDIA 4, 20133 Milan, Italy; 12 Sanofi, 20158 Milan, Italy; mattia.battistini@sanofi.com (M.B.); eleonora.cantoni2@sanofi.com (E.C.); filippo.cipriani@sanofi.com (F.C.); giovanna.sala@sanofi.com (G.S.); annalisa.stassaldi@sanofi.com (A.S.); 13 Neuroimmunology Unit, Santa Lucia Foundation IRCCS, 00179 Rome, Italy; martapirronello96@gmail.com; 14 FederASMA e ALLERGIE Federazione Italiana Pazienti ODV, 59100 Prato, Italy; 15 Otorhinolaryngology-Head and Neck Surgery, A. Gemelli Hospital Foundation IRCCS, 00168 Rome, Italy; eugenio.decorso@policlinicogemelli.it

**Keywords:** chronic rhinosinusitis with nasal polyps, disease burden, symptoms, patient journey, emotional burden, everyday living, caregiver burden, productivity loss, snoring

## Abstract

**Background:** Chronic rhinosinusitis with nasal polyps (CRSwNPs) is a chronic inflammatory disease associated with frustrating symptoms, particularly nasal obstruction and loss of smell. We conducted a patient survey on the significant burden of the disease, with a specific focus on conditions that affect health, sleep quality, absenteeism, and presenteeism, including the caregivers’ perspectives. **Methods:** An online questionnaire was sent to 4230 randomly selected recipients, and 200 matched the inclusion criteria for self-reported CRSwNPs symptoms. A total of 100 participants not matching the inclusion criteria for CRSwNPs were recruited as a control group. The study also collected the perspectives of 50 caregivers. **Results:** Patients with CRSwNPs experienced very bothersome symptoms, such as nasal congestion, headache, and rhinorrhoea, with a profound impact on their health-related quality of life (HRQoL). The patients and their caregivers showed significantly lower quality of sleep, experiencing a poor night’s sleep on average 72.1 and 51.7 days per year, respectively. Smell and taste impairments significantly impacted patients’ social and working lives, with 39.5% feeling in danger because of hyposmia and 34.5% because of limited taste. Out-of-pocket costs were up to EUR 40/month for 68.5% of patients. CRSwNPs alone was responsible for an average of 24.7 days of absenteeism and 25.1 days of presenteeism. **Conclusions:** Our results highlight how CRSwNPs has a negative impact on patients’ and caregivers’ HRQoL. Most bothersome and health-conditioning symptoms involve nose symptoms and poor sleep quality, resulting in patient absenteeism and presenteeism with a strong burden on cognitive and emotional functioning for both patients and their caregivers.

## 1. Introduction

Chronic rhinosinusitis (CRS) is a heterogeneous and multifactorial disease characterised by persistent inflammation of the nose and paranasal sinuses, which causes nasal obstruction, nasal discharge, facial pain, and smell disturbance [1]. The duration of the sinonasal inflammation must exceed 12 weeks for the disease to be defined as chronic [2]. The disease’s aetiology involves immune and epithelial components, influenced by the microbiome, environment, and genetics [3].

Chronic rhinosinusitis with nasal polyps (CRSwNPs) is diagnosed based on subjective and objective signs of sinonasal inflammation with inflammatory changes of the sinonasal mucosa [4]. Approximately 25–30% of all CRS patients present nasal polyps [4]. The prevalence of CRSwNPs is thought to be around 1.1% in the USA and between 2.1% and 4.4% in Europe. CRSwNPs is closely associated with asthma [3], as shown in a recent study where about 42% of Italian patients with severe asthma also presented CRSwNPs [5]. The disease typically manifests around the age of 42, with diagnosis usually occurring later, up to age 60 [4].

Although nasal polyps may be associated with different endotypes, in Western countries, type 2, which is characterised by the accumulation of eosinophils, mast cells, basophils, T-helper 2 cells (Th2), and the production of type 2 cytokines (interleukin (IL) 4, IL-5, and IL-13) has high prevalence. This endotype is frequently associated with a high risk of recurrences [1].

The key symptoms of CRSwNPs are complete or partial loss of smell (anosmia or hyposmia), nasal obstruction or congestion, nasal secretion, postnasal drip, and facial pain or pressure [1], which significantly reduce the HRQoL of both patients and caregivers [6]. A recent systematic review highlights a gap in research on the burden of CRSwNPs on caregivers, suggesting that this is an area worth exploring [7].

The current treatment of CRSwNPs is based on saline nasal irrigation, intranasal and/or systemic steroids, and endoscopic sinus surgery [8]. Systemic steroids are advised only in short-term cycles, but not for long-term use due to possible side effects [9,10]. Surgery remains the standard treatment to unblock nasal cavities, despite its temporary benefits due to the high incidence of relapse [11]. Targeted biologic therapies have only recently been available in the USA and Europe for patients with CRSwNPs. Although many different management options exist, there is no definitive cure for the disease [12].

The healthcare costs associated with rhinosinusitis are significant. In the USA, managing CRS costs USD 2609 per patient annually [8]. Data are still scarce in Europe, but a German study reported costs of EUR 1501 per patient per year, mainly due to outpatient visits and hospitalisation [13]. Indirect costs are even more relevant, with EUR 5659 per patient/year, largely due to productivity losses (presenteeism) and absenteeism [13]. Nasal congestion leads to poor sleep, daytime drowsiness, and ultimately limited productivity or missed workdays [14].

Very little is known about the daily impact of living with CRSwNPs, particularly how sleep disturbance (quality of sleep and number of restless nights) translates into reduced productivity. There is a need for more data on the experiences of caregivers living with patients who have the disease. This study aimed to quantify the burden of CRSwNPs on both patients and their caregivers/families. It focused on identifying unmet needs, particularly regarding sleep quality and its impact on daily functioning.

## 2. Materials and Methods

### 2.1. Study Design

The sample used in the survey was drawn from the Doxa Population Panel, a proprietary, quality-assured database with more than 140,000 entries representing the Italian population from a socio-demographic perspective. Data were collected using computer-assisted web interviews (CAWI) performed with a semi-structured questionnaire sent randomly by email to 4230 subjects. The questionnaire consisted of two sections. The first section (screening) was aimed to assess whether the respondents were eligible for the study (patients or caregivers), whereas the second section contained the research questions, based on a review of the relevant literature. The questionnaire included open questions, 5-point Likert-scale questions, multiple-choice questions, closed questions (yes/no), and evaluation scale questions (e.g., 1 to 10). Data were gathered in October 2022. This study complied with the STROBE cross-sectional reporting guidelines [15].

### 2.2. Questionnaire

The questionnaire covered three main aspects of living with CRSwNPs. These included the patient journey (diagnosis, symptoms, comorbidities, disease history, disease management, direct patient costs, and treatment satisfaction), HRQoL (sleep quality, reliability of sense of smell and taste, emotional burden, and impact on daily living, including work and school), and awareness of supporting initiatives for patients with CRSwNPs. The full questionnaire is reported in the Supplementary Material.

### 2.3. Study Population

Inclusion criteria were the presence of either nasal congestion/obstruction or nasal discharge in the previous 6 months, in addition to another symptom such as reduced sense of smell or facial pain. A second confirmatory inclusion criterion was a diagnosis of CRSwNPs by a healthcare professional (HCP). Accordingly, caregivers were enrolled by asking whether their partner had been diagnosed with CRSwNPs by an HCP. Patients in which the diagnosis of CRSwNPs was excluded by an HCP were considered controls. Finally, patients currently being treated with biologics were excluded. The first 200 respondents who met the exclusion criteria were enrolled in the patient group. In parallel, the first 100 people without symptoms and a diagnosis of CRSwNPs were enrolled as controls.

### 2.4. Statistical Analysis

Statistical analyses were performed using the pTabs2 software (version 2.7.27). Categorical variables were compared using the χ^2^ test. Nonparametric Spearman correlation tests were used for continuous variables. Statistical significance was set at *p* ≤ 0.05 (two-tailed).

## 3. Results

### 3.1. Demographics of the Study Population

The questionnaire was sent to 4230 random recipients and was completed by 927 respondents. Applying predefined inclusion and exclusion criteria, the final study population consisted of 200 adult patients (≥18 years) with CRSwNPs, 100 individuals without CRSwNPs (control group), and 50 caregivers of patients with CRSwNPs. The mean age was 43 years amongst patients with CRSwNPs and 51 years in the control group. Female respondents represented 56% of patients with CRSwNPs (52% in the control group). The caregiver group consisted of partners (48% of female and 52% of male patients). Demographic and comorbidity data are summarised in Table 1 and in Figure S1.

Overall, CRSwNPs patients had significantly more comorbidities than the control group (3.5 versus 1.5; *p* ≤ 0.05). Although pollen and dust allergies were the most frequent conditions (49.4% and 47%, respectively), mostly related to type 2 inflammation such as atopic dermatitis and allergic conjunctivitis, 34.5% of patients with CRSwNPs had concomitant asthma, equally distributed between mild and moderate/severe asthma.

Similar patterns of comorbidity were also found in CRSwNPs family members, such as parents, siblings, grandparents, uncles, and cousins (2.8 vs. 1.4; *p* ≤ 0.05). CRSwNPs, asthma, atopic dermatitis, and eosinophilic esophagitis were significantly more prevalent in the families of the CRSwNPs patients compared to the non-CRSwNPs control group (Table 1).

### 3.2. Symptoms

**Figure 1:**
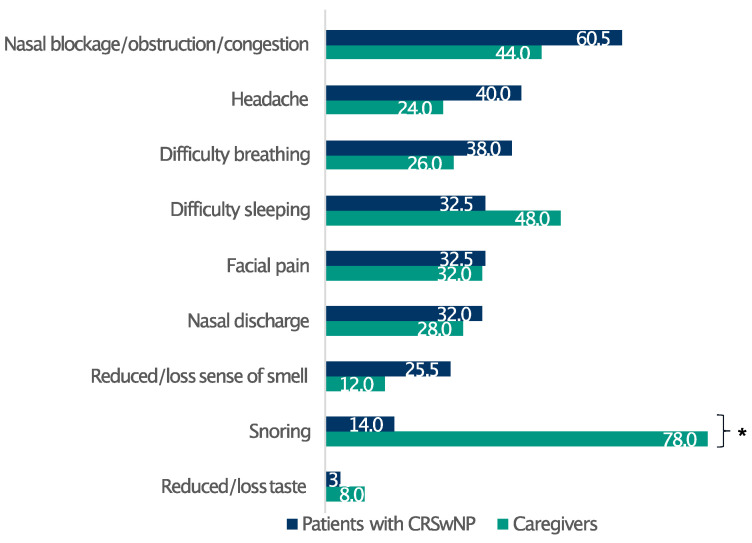
Patients’ and caregivers’ perceptions of CRSwNPs symptom burden. Data are expressed as percentages. * Statistically significant (*p* ≤ 0.05).

Overall, 83.5% of patients had severe CRSwNPs (SNOT-22 score > 50), whereas only 2.5% of the respondents had mild disease (SNOT-22 score 8–20) [16] (Table 1). The respondents reported a broad spectrum of discomfort, prominently featuring a blocked nose, nasal obstruction/congestion, and nasal discharge (Figure S2). Furthermore, the survey identified the most bothersome symptoms among patients with CRSwNPs and their relatives. Remarkably, whereas nasal congestion/obstruction, headache, and difficulty breathing profoundly affected patients’ well-being, the most relevant issue for their relatives emerged as difficulty in sleeping, principally due to patients snoring at night (Figure 1).

### 3.3. Sleep Impairment and Daily Drowsiness

**Figure 2:**
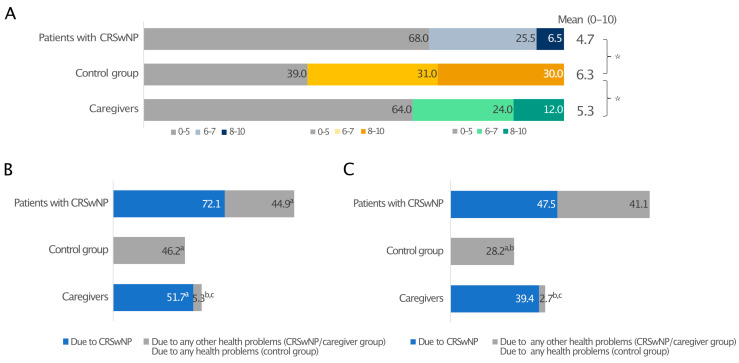
Sleep quality in patients with CRSwNPs, controls, and the CRSwNPs caregivers’ group. Data are expressed as percentages (**A**). The frequency of days of poor sleep quality (**B**) and daytime drowsiness (**C**) due to CRSwNPs or any other health problems. For data in A, 0 indicates the worst-possible quality of sleep, and 10 indicates the best-possible quality of sleep. * Statistically significant (*p* ≤ 0.05). In panels B and C, ^a^, ^b^, and ^c^ indicate statistical significance versus CRSwNPs in patients with CRSwNPs, any other health problems in patients with CRSwNPs, or the control group, respectively (*p* ≤ 0.05).

When quality of sleep (QoS), defined as difficulty falling asleep, frequent waking, and having difficulty falling back asleep after waking, during the previous two weeks (0—the worst possible quality of sleep and 10—the best possible quality of sleep) was reported, patients with CRSwNPs and their caregivers awarded suboptimal and similar mean scores to their sleep quality, 4.7 and 5.3, respectively. In both cases, these were significantly lower than the control group (6.3; *p* ≤ 0.05) (Figure 2A).

When nights with QoS disturbances due to CRSwNPs or any other health-related issue and days with drowsiness over the past 12 months were investigated, CRSwNPs had a considerable impact on the sleep of both patients and caregivers, who experienced a poor night’s sleep on an average of 72.1 days/year and 51.7 days/year, respectively. CRSwNPs alone caused more sleepless nights than any other health problem (44.9 days/year; *p* ≤ 0.05), which is very close to the 46.2 days/year measured for all health problems in the control group without CRSwNPs (Figure 2B). A prolonged lack of good-quality sleep resulted in daytime drowsiness in both patients and caregivers, with 47.5 and 39.4 days/year of daytime drowsiness due to CRSwNPs, respectively, whereas control individuals had significantly better sleep quality (Figure 2C).

### 3.4. Patient Journey

The history of the disease, including diagnosis and cure, showed that patients had suffered from CRSwNPs for 11.3 years on average. Patients often experienced a diagnostic delay, with an average of 7.2 years from presenting the first symptoms and the recognition of CRSwNPs by the HCP (Table 2). Most diagnoses (62%) were made by an ear, nose, and throat (ENT) specialist, who was also the first specialist patients consulted when symptoms appeared (52%). The second-most frequent physician involved with the diagnosis and follow-up was the general practitioner (GP). The distribution of specialists currently involved in patient care was similar to that of HCPs responsible for the diagnosis and those who were consulted when the symptoms first appeared. It is worth noting that at the time of the survey, 12.5% of patients were not followed up by any specialist (Table 2).

More than half (53.1%) of the patients were on active pharmacological treatment at the time of the survey (Table 3). Among patients with CRSwNPs, the average treatment duration exceeded seven years. Table 3 shows the drugs used in the previous 6 months, highlighting nasal sprays containing cortisone as the most frequently used treatment for CRSwNPs. These medications were predominantly prescribed by ENT specialists and GPs.

**Figure 3:**
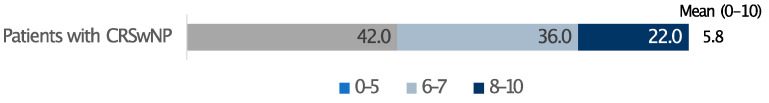
Satisfaction level with the cost/benefit ratio of the treatment. 0 indicates not at all advantageous and 10 very advantageous. Data are expressed as percentages.

In terms of out-of-pocket expenses for CRSwNPs therapies, 68.5% of patients reported monthly costs of up to EUR 40/month, while one in four patients indicated spending between EUR 40 and EUR 80 per month (Table 3). The overall treatment satisfaction, evaluated on a scale from 0 to 10 and accounting for both cost and effectiveness, averaged 5.8 (Figure 3).

### 3.5. Emotional Burden and Daily Limitations

**Figure 4:**
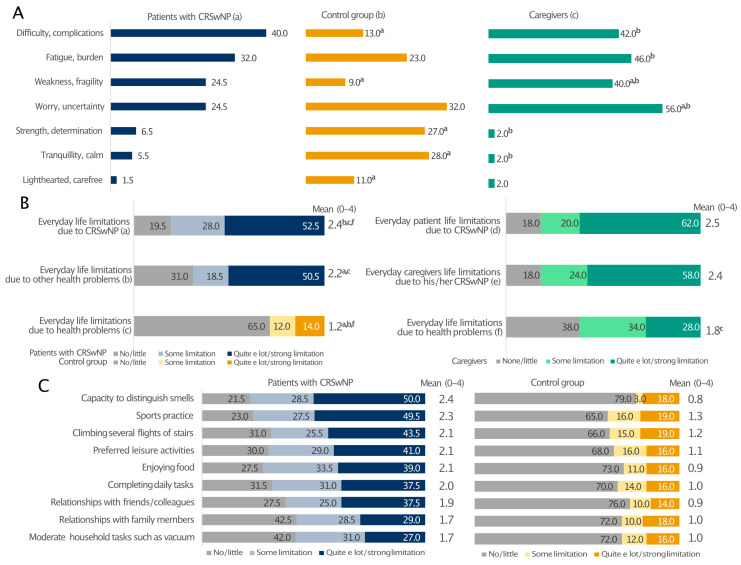
Prevalent emotions (**A**) and limitations to everyday life due to CRSwNPs or other health problems (**B**) and regarding various everyday life activities (**C**) in patients with CRSwNPs, their caregivers, and the control group. All analyses in panel (**C**) comparing patients with CRSwNPs to the control group were statistically significant (*p* ≤ 0.05). Panel (**A**): a, patients with CRSwNPs; b, control group; c, caregivers. ^a^, ^b^, and ^c^ indicate statistical significance versus a, b, c (*p* ≤ 0.05). Panel (**B**): a, b patients with CRSwNPs; c, control group; d–f, caregivers. ^a^, ^b^, ^c^ and ^f^ indicate statistical significance versus a, b, c, d or f (*p* ≤ 0.05). Data are expressed as percentages.

Patients with CRSwNPs and their caregivers expressed similar feelings about the disease (Figure 4A). Their prevalent emotions were the perception of difficulty/complications (40% and 42%), fatigue/burden (32% and 46%), and weakness/fragility (24.5% and 40%). The most frequent feeling among the relatives was the sense of worry/uncertainty (56% vs. 24% of patients; *p* < 0.05). In these two groups, the percentages of individuals feeling strength/determination, serenity/freedom, tranquillity/calm and being light-hearted/carefree were generally low. On the contrary, these emotions were more often encountered in the control group, with only 13% and 9% reporting difficulty/complications and weakness/fragility, respectively (Figure 4A). Moreover, the patients’ everyday life was limited by CRSwNPs and other health issues to a greater extent than in the control group (Figure 4B). Interestingly, CRSwNPs, unlike other health problems, limited the everyday life of caregivers as much as their partners (on average 2.4 and 2.5 on a 0–4 scale, relatively). Everyday life limitation of patients due to CRSwNPs was also perceived at the same level by both patients and caregivers (Figure 4B). In addition, all investigated patients’ daily activities were affected, particularly the capability to distinguish smell and the limitation in sports practice, which had the greatest impact on patients with CRSwNPs and not the control individuals (Figure 4C). This is consistent with the fact that CRSwNPs patients reported significantly lower confidence in smell (15.5% vs. 74%; *p* ≤ 0.05; Figure S3A) and taste (29% vs. 85%; *p* ≤ 0.05; Figure S3B) than the control group. These sensory impairments also had a significant impact on patients’ social and professional lives (Figure S3C). Overall, 39.5% of CRSwNPs patients felt in danger because of hyposmia and 34.5% because of poor taste (e.g., risk of eating spoiled food), whereas only a few healthy people felt the same way (Figure S3D,E; *p* ≤ 0.05). Moreover, Pearson’s correlation analysis showed that all everyday life limitations positively correlated with the diagnosis of CRSwNPs (*p* ≤ 0.001) (Supplementary Figure S4).

### 3.6. Quality of Life and Social/Couple Relationships

**Figure 5:**
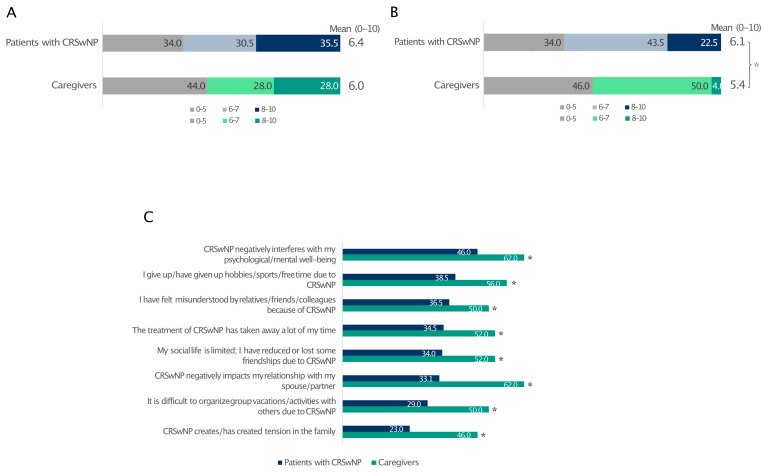
Impact of CRSwNPs on the level of stress reported by patients and caregivers (**A**) and how CRSwNPs affects their quality of life (**B**). The level of agreement (quite/very) with statements related to couple relationships and social life (**C**). For data in A and B, 0 indicates the least impact and 10 is the greatest impact. * Statistically significant compared to patients with CRSwNPs (*p* ≤ 0.05). Data are expressed as percentages.

The level of stress was similar between patients affected by CRSwNPs and their partners (66% and 56%, respectively) (Figure 5A). Despite this, CRSwNPs negatively interfered with patients’ quality of life more than that of their partners (Figure 5B). In contrast, caregivers reported a much worse impact of CRSwNPs on their social/couple life, pointing out how CRSwNPs negatively interfered in the relationship with their partners, creating tensions within the family circle (Figure 5C).

### 3.7. Working/School Life

**Figure 6:**
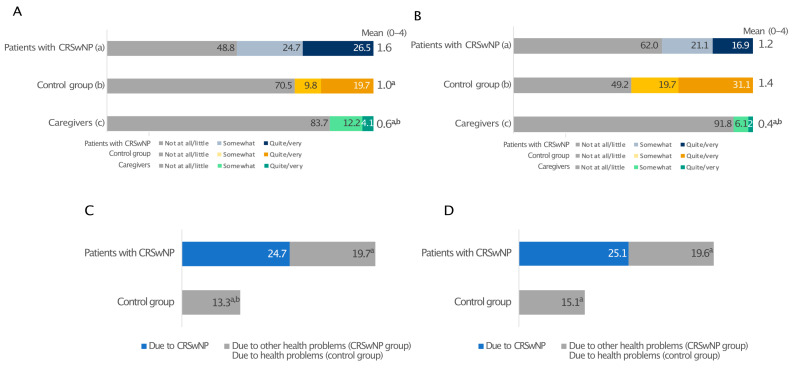
Impact of health on working life reported by patients with CRSwNPs, controls, and the relative group. The level of agreement with the statements “I do not work/study serenely” (**A**) and “I had to forgo some work/study opportunities” (**B**). Days of absenteeism (**C**) and presenteeism (**D**). Panel (**A**,**B**): a, patients with CRSwNPs; b, control group without CRSwNPs; c, relatives of CRSwNPs patients. ^a^ and ^b^ indicate statistical significance versus a, b, c (*p* ≤ 0.05). Panels (**C,D**): ^a^, ^b^ indicate statistical significance versus CRSwNPs in patients with CRSwNPs, and versus other health problems (CRSwNPs group), respectively (*p* ≤ 0.05). Data are expressed as percentages.

People with CRSwNPs generally carried out their work and school activities less comfortably than those without CRSwNPs (Figure 6A). CRSwNPs prevented every second patient from carrying out their work/school activities (Figure 6A) and triggered forgoing some work/study opportunities in 4 out of 10 cases (Figure 6B). Interestingly, very few caregivers (16.3%) reported issues with work activities (Figure 6A) and rarely (8.1%) had to give up work opportunities (Figure 6B).

In investigating absenteeism and presenteeism over the last 12 months, we calculated the number of days missed and with decreased productivity due to a lapse in attention, either at work or at school/university, directly related to CRSwNPs, to any other health issues (CRSwNPs group), and to any health-related reasons (control group) (Figure 6C,D).

People without CRSwNPs experienced 13.3 days of absenteeism and 15.1 days of presenteeism (equal to 28.4 days of total work impairment). In contrast, CRSwNPs alone was responsible for 49.8 days per year of work/study impairment, almost equally divided between presenteeism and absenteeism. Moreover, 73% of patients agreed with the statement that physical pain provoked by CRSwNPs negatively affects work and studies (Figure S5).

### 3.8. Unmet Patient Needs

Over 90% of patients had never heard of public initiatives to promote the awareness and knowledge of CRSwNPs. Patients reported the need for curative, less invasive and costly therapies (22%), access to specialist treatment (5.5%), and more initiatives to inform them and the public, including discoveries concerning the disease and its treatments (10%). Finally, patients expressed interest in psychological and economic support and welfare and information concerning the right to leave of absence because of their illness (25%).

## 4. Discussion

This study exposed the significant daily burden of CRSwNPs in both patients and caregivers.

The patient journey revealed a 7.2-year delay between symptom onset and confirmatory diagnosis of CRSwNPs. This was longer than the 3 to 5 years reported elsewhere [17]. Delayed diagnosis leads to inappropriate and ineffective treatments, while successful treatment improves HRQoL in patients with CRSwNPs [6]. One patient out of three had comorbid asthma, and other type 2 comorbidities, such as atopic dermatitis, were also quite distributed among their family circle [18,19]. Despite more than 80% of survey participants having a severe form of CRSwNPs, only 20% underwent surgery, while 12.5% of patients were not in follow-up by any specialist. More surprisingly, roughly half were not taking any medications. We speculate that this may be explained by the low perceived efficacy and relatively high out-of-pocket cost of therapies, as also documented by Luke et al. [20], in which oral steroids and surgery were perceived as only temporarily effective.

In agreement with previous papers, sinonasal and sleep symptoms were reported as the most bothersome manifestations. Poor sleep quality is an important aspect of the disease burden [1]. In our study, about 70% of the CRSwNPs patients reported poor sleep quality. Ferri et al. obtained similar data in their study [21]. In particular, polyp size may correlate with symptoms from the sleep SNOT-22 subdomains [22], and the impact of CRSwNPs on sleep is greater in patients with poorly controlled disease [23]. Cytokines and their receptors are key in sleep physiology [24]. IL-1β and tumour necrosis factor (TNF) α regulate sleep stages [24,25]. IL-4, IL-13, and transforming growth factor (TGF) β, which are upregulated in CRS, antagonise IL-1β and TNF-α, thereby reducing sleep [26,27].

Olfactory and gustatory dysfunction affects HRQoL in patients with CRSwNPs [6,28]. Patients with CRSwNPs in our study reaffirmed an inability to discriminate between smells and tastes as an important contributor to the overall burden of disease [6,28]. Patients with anosmia reported reduced enjoyment of food, difficulties with cooking or assessing personal hygiene, and an inability to detect hazards. Reduced smell and taste affected patients’ social and professional lives and contributed to a feeling of danger due to olfactory and gustatory dysfunctions.

In recent years, the importance of recognising the burden of caregivers in assisting patients with chronic diseases has emerged [29]. Constant caregiving affects relatives in various ways, including the ability to work, socialise, and maintain good health, and it is a key part of home-based long-term care [30]. A recent study found that caregivers felt more burdened as the QoL of patients with CRS declined, and their perceptions were even worse than those reported by patients [31]. There is still a profound lack of knowledge of the real burden of CRSwNPs on caregivers. This survey shows that poor sleep quality is shared between patients and caregivers. Both had similar sleep quality scores and comparable daytime drowsiness. While patients found nasal symptoms most bothersome at night, their caregivers underlined how their spouses’ snoring was the main cause of their poor sleeping. Patients’ snoring resulted in difficulty sleeping at night and daytime tiredness for caregivers.

In parallel, CRSwNPs burdens the cognitive and emotional functioning of patients and their caregiving relatives. Coping with CRSwNPs is as stressful for carers as it is for patients, leading to withdrawal from social life and couple conflict [6].

Little literature is available on work/school implications and missed career opportunities for patients with CRSwNPs. Additionally, the limited results reported are not always comparable, as they are highly dependent on disease severity and the proportion of patients with and without nasal polyps. Sleep disturbance is linked to reduced productivity, with presenteeism being the most affected [32]. Depression is also associated with days lost from work or school due to CRS [33]. Interestingly, data from our survey show that a limited percentage of relatives mention having some issues with work activities and work opportunities.

The present study has some limitations. First, all observations were based on respondents’ self-reporting. Despite trying to limit recall bias in our questionnaire by asking questions about the last 2 weeks to 6 months, that risk cannot be excluded. Moreover, online surveys require a certain degree of computer literacy, which may inevitably cause a selection bias, as persons incapable of or uncomfortable with using computers might not participate in such a survey. We also recognise that sending the survey to a population database, albeit representative of the whole of Italy, may result in potential bias. Finally, we excluded patients undergoing treatment with novel biologic molecules so that we could evaluate the actual limitations of CRSwNPs management before the widespread availability of new and more specific treatments, which currently still have very limited access in Italy. Our results need to be updated when their adoption becomes more disseminated. We are aware of the exploratory nature of our work and hope that this will stimulate further interest in the subject and encourage future research.

## 5. Conclusions

In conclusion, our study highlights the significant burden of CRSwNPs on Italian patients. From the disease management perspective, patients advocate for better therapeutic options to improve their quality of life as well as the quality of life of their families.

Notably, although exploratory, the study also examined the impact on caregivers, particularly partners, and found that CRSwNPs indirectly affects partners as much as patients, leading to shared challenges such as sleep deprivation and emotional distress, framing CRSwNPs as a “couple’s” disease.

Awareness campaigns might activate patients earlier and contribute to reducing diagnostic delay. In parallel, policymakers, scientific societies, and patient advocacy groups might encourage a more integrated and multidisciplinary approach among healthcare professionals.

## Figures and Tables

**Table 1:** Demographics and clinical situation of patients with and without CRSwNPs.

	Patient Group (n = 200)	Control Group (n = 100)
**Age (years)**	43	51
**Female/male (%)**	56/44	52/48
**Severity of CRSwNPs (%)**		
Mild (SNOT-22 score 8–20)	2.5	NA
Moderate (SNOT-22 score >20–50)	14.0	
Severe (SNOT-22 score >50)	83.5	
Mean SNOT-22	6.89	
**CRSwNPs and asthma (%)**		
CRSwNPs with mild asthma	17.5	NA
CRSwNPs with moderate asthma	12.5	
CRSwNPs with severe asthma	5.0	
CRSwNPs with other condition/s ^	3	
**Mean number of past and current comorbidities**	3.5	1.5
**Past and current comorbidities (%)**		
Pollen allergies *	49.5	16.0
Dust allergies *	47.0	16.0
Allergic conjunctivitis *	32.5	13.0
Food allergies *	30.0	12.0
Hay fever *	25.0	7.0
Atopic dermatitis *	24.5	11.0
Pet fur allergies *	22.5	7.0
Severe eating disorders *	7.5	1.0
**Comorbidities in the family circle (%)**		
Plant and pollen allergies *	52.0	30.0
Dust allergies *	48.0	26.0
Asthma *	36.0	18.0
CRSwNPs *	30.5	2.0
Atopic dermatitis *	28.0	10.0
Eosinophilic esophagitis *	16.0	3.0
Insect bite allergies *	15.5	4.0
None of the above *	19.0	38.0

CRSwNPs, chronic rhinosinusitis with nasal polyps; NA, not applicable. ^ Chronic rhinosinusitis with nocturnal apnoea, chronic rhinosinusitis with turbinate hypertrophy, chronic rhinosinusitis with chronic obstructive pulmonary disease. * Statistically significant (*p* ≤ 0.05).

**Table 2:** The patient journey from symptoms to diagnosis.

	Patient Group (n = 200)		
**Mean disease duration (years)**	11.3		
**Mean time from first symptoms to diagnosis (years)**	7.2		
**Specialist (%)**	**Consulted with first symptoms**	**Made diagnosis**	**Currently under specialist management**
ENT	52.0	62.0	53.5
GP	27.0	19.5	26.0
Allergologist	11.0	11.5	10.0
Chest physician	4.0	5.0	7.0
Immunologist	1.0	1.0	2.0
Did not remember	5.0	1.0	12.5 ^

ENT, ear, nose, and throat specialist; GP, general practitioner. ^ Currently not managed by any specialist.

**Table 3:** The patient journey regarding treatment.

	Patient Group (n = 200)	
**Past and current nasal irrigations (%)**		
Often	52.0	
Rarely	29.7	
Never	18.3	
**Currently in pharmacological treatment, yes/no (%)**	53.1/46.9	
**Mean duration of pharmacological treatment (years)**	7.3	
**Pharmacological treatment (%)**	**In the last 6 months (n = 175)**	**Previously**
Corticosteroid spray	55.4	65.7
Oral corticosteroids	20.0	41.1
Injectable corticosteroids	5.8	10.3
Other ^	5.8	9.0
No pharmacological treatment	13.7	NA
**Past use of biologics (%)**	12	
Benralizumab	5.5	
Mepolizumab	5.5	
Omalizumab	3.0	
Dupilumab	1.5	
**Specialist who prescribed pharmacological treatment in the last 6 months (%)**		
ENT	63.2	
GP	17.1	
Allergologist	9.9	
Chest physician	6.6	
Immunologist	1.3	
Other	1.4	
Unknown	0.7	
**Mean monthly spending on treatments (%), EUR**		
0–10	31.0	
11–40	37.5	
41–80	26.5	
>80	5	

ENT, ear, nose, and throat specialist; GP, general practitioner; NA, not applicable. ^ Antibiotics, antihistamine agents, decongestants, non-steroidal anti-inflammatory drugs.

## Data Availability

All data are available in the Supplementary Materials file.

## Supplementary Materials

The following supporting information can be downloaded at https://www.mdpi.com/article/10.3390/healthcare13040430/s1. Figure S1: Geographical (A) and age (B) distribution of patients with CRSwNPs, their relatives, and control individuals; Figure S2: Results from the SNOT-22 questionnaire related to symptoms that occurred in the previous two weeks; Figure S3: The relationship with the sense of smell and taste: reliability of sense of smell (A) and sense of taste (B), impact of sense of smell and of sense of taste on social and working life (C), perception of danger due to the deficiency of sense of smell (D) or smell of taste (E). For data in A and B, 0 indicates not reliable at all, and 10 indicates extremely reliable. All comparisons in panels C, and those marked by * are statistically significant (*p* ≤ 0.05). Data are expressed as percentages; Figure S4: Pearson correlation coefficient between limitations and aspects and activities of daily life in patients with CRSwNP. All correlations are statistically significant, (*p* ≤ 0.001). Figure S5: Consequences of physical pain on work and study in patients with CRSwNPs.
