# Supplementary material for: The Impact and Burden of Chronic Rhinosinusitis with Nasal Polyps on Patients and Their Family Caregivers: A Nationally Representative Survey
Source: Healthcare (Basel). 2025 Feb 17;13(4):430. doi: 10.3390/healthcare13040430 (PMC11855350; doi:10.3390/healthcare13040430)

Supplementary Figure S1. Geographical (A) and age (B) distribution of patients with CRSwNP, their relatives and control individuals.

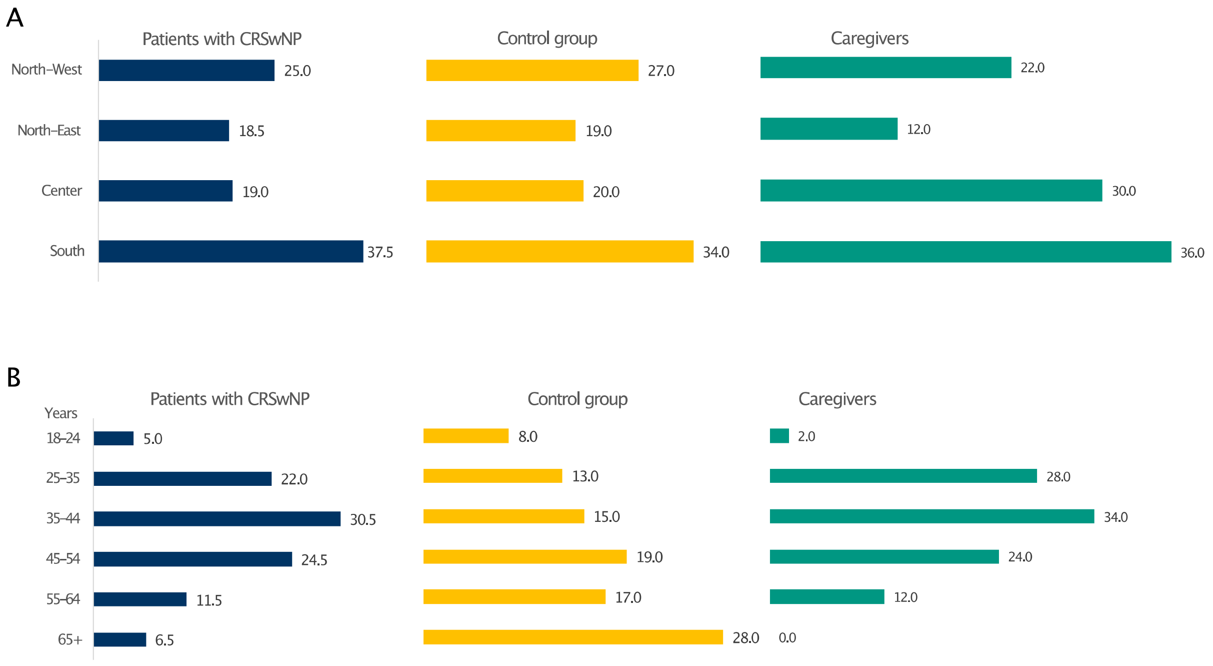

Supplementary Figure S2. Results from the SNOT-22 questionnaire related to symptoms that occurred in the previous two weeks.

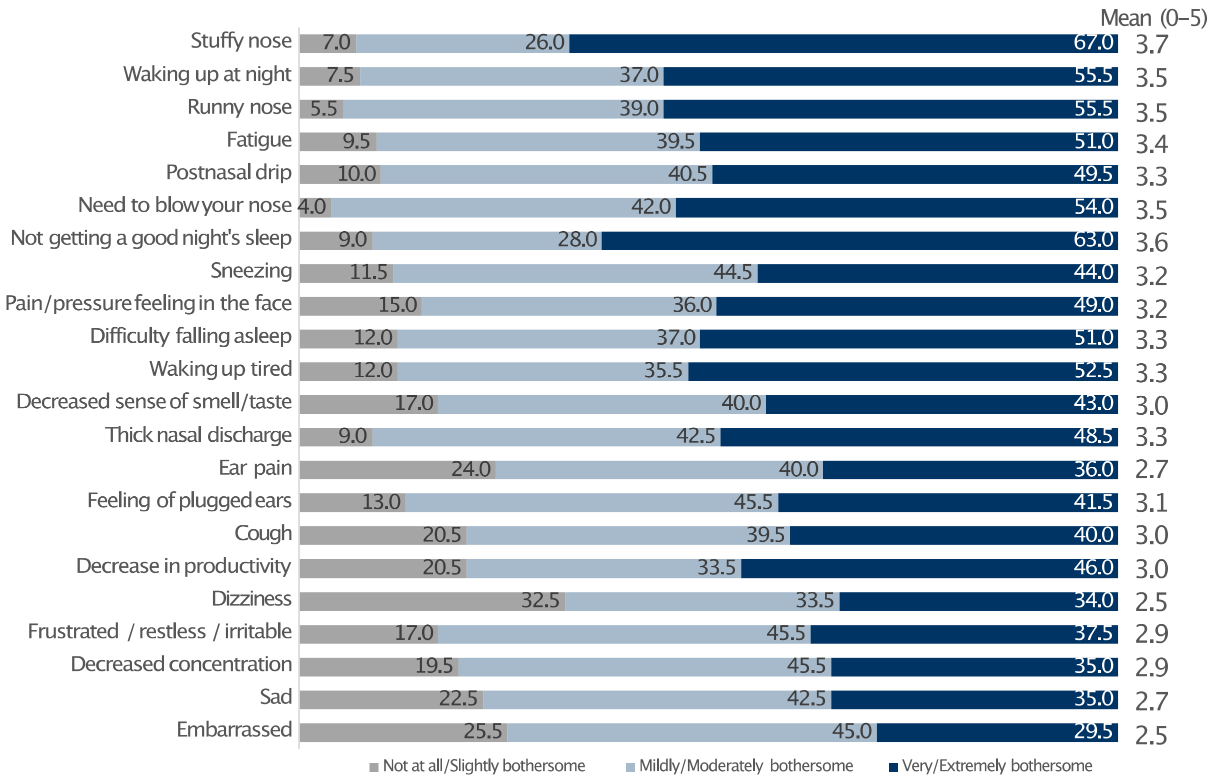

Supplementary Figure S3. The relationship with the sense of smell and taste: reliability of sense of smell (A) and sense of taste (B), impact of sense of smell and of sense of taste on social and working life (C), perception of danger due to the deficiency of sense of smell (D) or smell of taste (E). For data in A and B, 0 indicates not reliable at all, and 10 indicates extremely reliable. All comparisons in panels C, and those marked by * are statistically significant (p ≤ 0.05). Data are expressed as percentages.

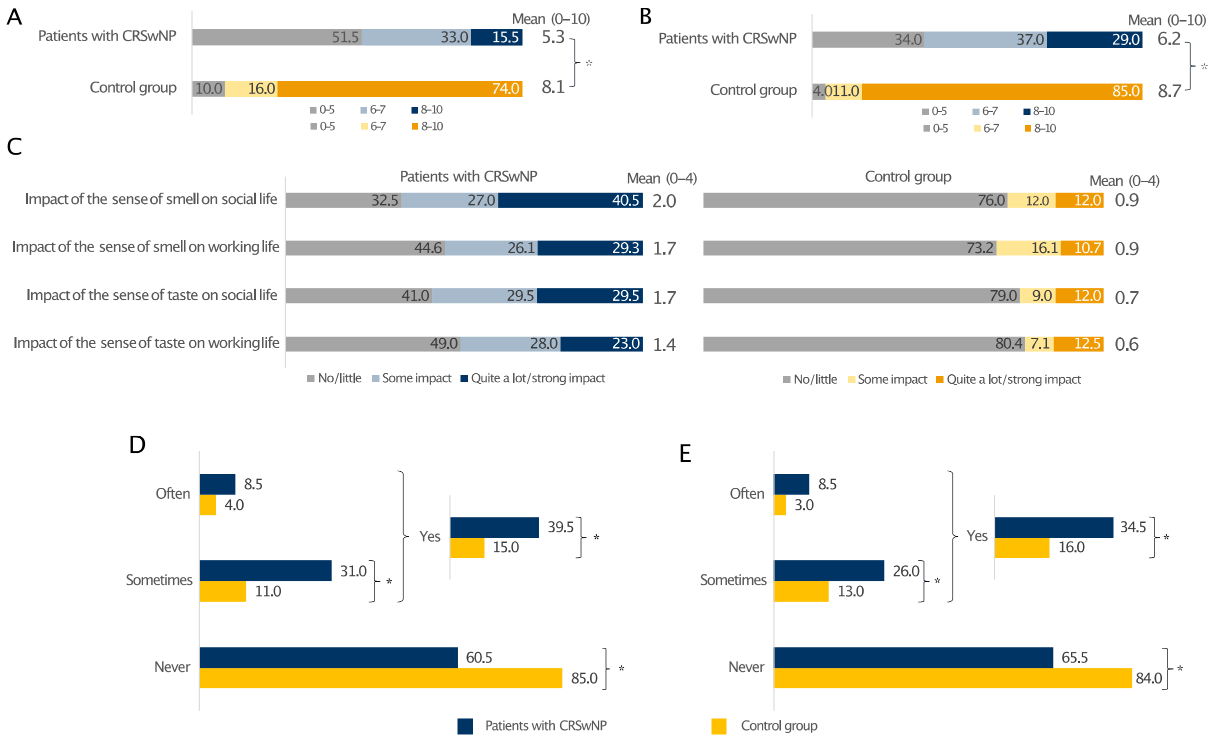

Supplementary Figure S4. Pearson correlation coefficient between limitations and aspects and activities of daily life in patients with CRSwNP. All correlations are statistically significant, ($p \leq 0.001$).

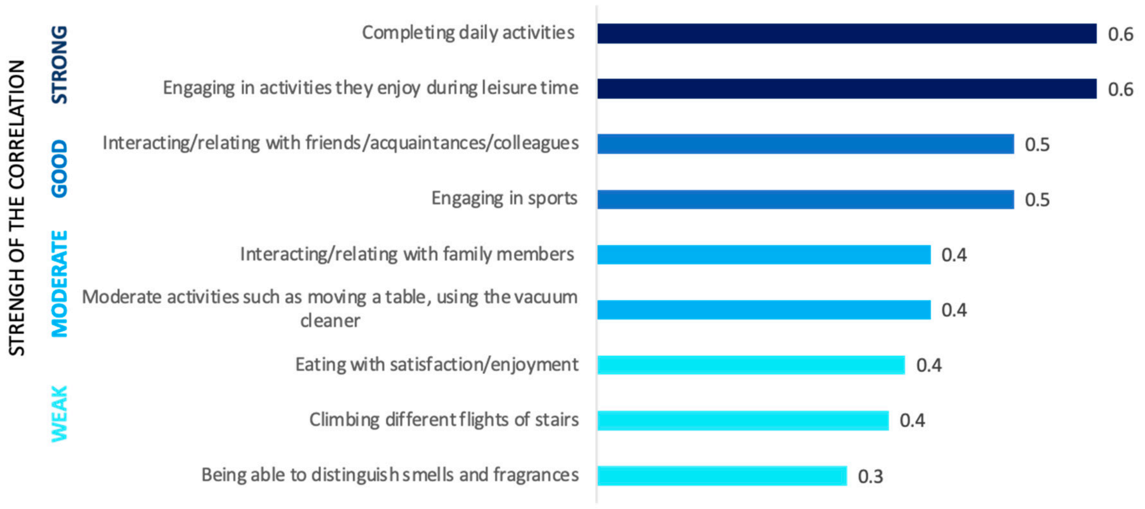

Supplementary Figure S5. Consequences of physical pain on work and study in patients with CRSwNP.

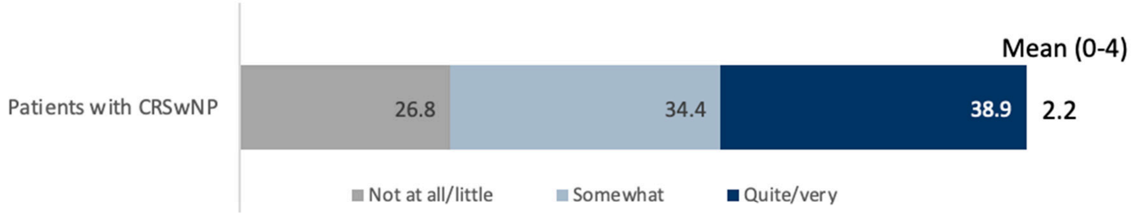

Supplement: Supplementary file 1 [file healthcare-13-00430-s001.zip › healthcare-3383762-supplementary.pdf]
